# FRAP Analysis Reveals Stabilization of Adhesion Structures in the Epidermis Compared to Cultured Keratinocytes

**DOI:** 10.1371/journal.pone.0071491

**Published:** 2013-08-19

**Authors:** Henry P. Foote, Kaelyn D. Sumigray, Terry Lechler

**Affiliations:** 1 Department of Cell Biology, Duke University Medical Center, Durham, North Carolina, United State of America; 2 Department of Dermatology, Duke University Medical Center, Durham, North Carolina, United States of America; 3 Department of Biology, University of North Carolina at Chapel Hill, Chapel Hill, North Carolina, United States of America; Northwestern University Feinberg School of Medicine, United States of America

## Abstract

Proper development and tissue maintenance requires cell-cell adhesion structures, which serve diverse and crucial roles in tissue morphogenesis. Epithelial tissues have three main types of cell-cell junctions: tight junctions, which play a major role in barrier formation, and adherens junctions and desmosomes, which provide mechanical stability and organize the underlying cytoskeleton. Our current understanding of adhesion function is hindered by a lack of tools and methods to image junctions in mammals. To better understand the dynamics of adhesion in tissues we have created a knock-in ZO-1-GFP mouse and a BAC-transgenic mouse expressing desmoplakin I-GFP. We performed fluorescence recovery after photobleaching (FRAP) experiments to quantify the turnover rates of the tight junction protein ZO-1, the adherens junction protein E-cadherin, and the desmosomal protein desmoplakin in the epidermis. Proteins at each type of junction are remarkably stable in the epidermis, in contrast to the high observed mobility of E-cadherin and ZO-1 at adherens junctions and tight junctions, respectively, in cultured cells. Our data demonstrate that there are additional mechanisms for stabilizing junctions in tissues that are not modeled by cell culture.

## Introduction

Cell-cell adhesions are critical structures for tissue formation and organ development. In addition to maintaining adhesion between adjacent cells, junctions play crucial roles in cell signaling, cell sorting and migration, and tissue barrier formation. Generally, cell-cell junctions are composed of transmembrane proteins that interact with adhesion proteins from neighboring cells, intracellular plaque or linker proteins, and proteins that anchor the junction to the cytoskeleton [1].

The three main types of cell-cell adhesions in epithelial cells – tight junctions, adherens junctions, and desmosomes – all follow this basic organization but differ in their distinct functions and components. Tight junctions play a critical role in the barrier function of a tissue, serving to prevent water loss and dehydration and to control the passage of ions and small molecules [2]. They are especially important in epithelia where they form a barrier between the body and the luminal compartments or the external environment. Adherens junctions are required for tight junction activity and play a role in ensuring mechanical stability of tissues [3], [4]. Desmosomes are plaque-like structures that are required for both mechanical stability and tissue integrity [5] as well as proper microtubule organization and adhesion development [6], [7]. They are especially important in tissues that experience extreme mechanical stress, such as the skin and the heart.

Although required for tissue stability, these junctions are not static structures, and much research has been done to study their regulation and the effect of structural changes on adhesion strength [8]. Studying protein turnover has provided a new path to understand the function of these junctions and what defects occur in various pathologies. For example, monitoring the turnover of desmosomal proteins has helped add to our understanding of the blistering disorder pemphigus [9]. Analysis of adherens junction dynamics has identified posttranslational modifications of E-cadherin that affect its turnover rate at the junction and which may be responsible for E-cadherin loss of function in different types of cancer [10]. Similarly, tight junction stability at the membrane can be altered through posttranslational modifications such as ubiquitination and phosphorylation, which have been shown to increase internalization of occludin, a transmembrane component at the tight junction [11].

Further studies have provided insight into the function of the adherens junction as a mechanosensor. Decreased junctional alpha-catenin mobility has been associated with increased cell tension and the increased binding of the actin-binding protein vinculin at the junction [12]. Additionally, the stability of E-cadherin may reflect the extent of its attachment to the actin cytoskeleton [13], [14]. In these studies, correlations have been made between junction strength and turnover, with the conclusion that less mobile junctional proteins provide stronger adhesion.

Clearly, understanding protein turnover can further our knowledge of junctions. However, prior work has predominantly addressed this issue in cultured cells. We are largely lacking a clear description of junction dynamics in mammalian tissues, due in part to the limited tools currently available for such analysis of adhesions. With the development of these tools, the study of junction dynamics in accessible tissues like the epidermis should be possible.

The epidermis is a stratified squamous epithelium that is amenable to live imaging of junctions in an intact tissue. In the epidermis, tight junctions are found only in the upper granular cells, a differentiated cell layer required for barrier function [15]. The tight junction protein zonula occludins 1 (ZO-1) serves as a key player in this barrier role, acting as a scaffolding protein to organize the tight junction structure and anchor it to the actin cytoskeleton [16]. Adherens junctions and desmosomes form at cell-cell contacts throughout the epidermis, in both the proliferative basal layer as well as the more differentiated upper layers, where they play an important adhesive role [3], [6]. E-cadherin is a homophilic transmembrane cadherin localized to epithelial adherens junctions [17]. An epidermis without cadherins fails to properly localize adherens junction components [3]. Desmoplakin (DP) anchors the desmosomal plaque to the intermediate filament cytoskeleton [6].

To probe junction dynamics and function in tissue, we have created two mice, a ZO-1-GFP knock-in mouse, and a DPI-GFP BAC transgenic mouse. The fluorescent tag allows for the observation of each protein at endogenous protein levels in tissue. Using these mice, as well as a previously described E-cadherin-CFP knock-in mouse [18], we examined three different types of cell-cell adhesions. We developed an imaging method for looking at junctions in the epidermis of embryonic mice and used Fluorescence Recovery After Photobleaching (FRAP) to observe the dynamics of adhesion proteins both within the architecture of a tissue and in cell culture. Surprisingly, we observed all three proteins to be remarkably stable in the epidermis, while cultured cells showed significant turnover of adherens and tight junctions.

## Results

### Characterization of ZO-1-GFP knock-in mouse

The development of reagents to visualize the dynamics of cell adhesion structures in cultured cells and in invertebrate organisms has been instrumental in our understanding of cell junction turnover and function. However, to date there are few mouse models to accomplish this in intact mammalian tissue. In order to observe the dynamics of tight junction-associated proteins in tissues, we created a ZO-1-GFP knock-in mouse. These mice express a carboxy-terminal tagged ZO-1-GFP from the endogenous ZO-1 promoter. We did not detect any overt phenotype in either heterozygous or homozygous mice. As loss of the tight junction scaffolding protein ZO-1 results in embryonic lethality [19], this demonstrates that the ZO-1-GFP knock-in is a functional allele.

**Figure 1:**
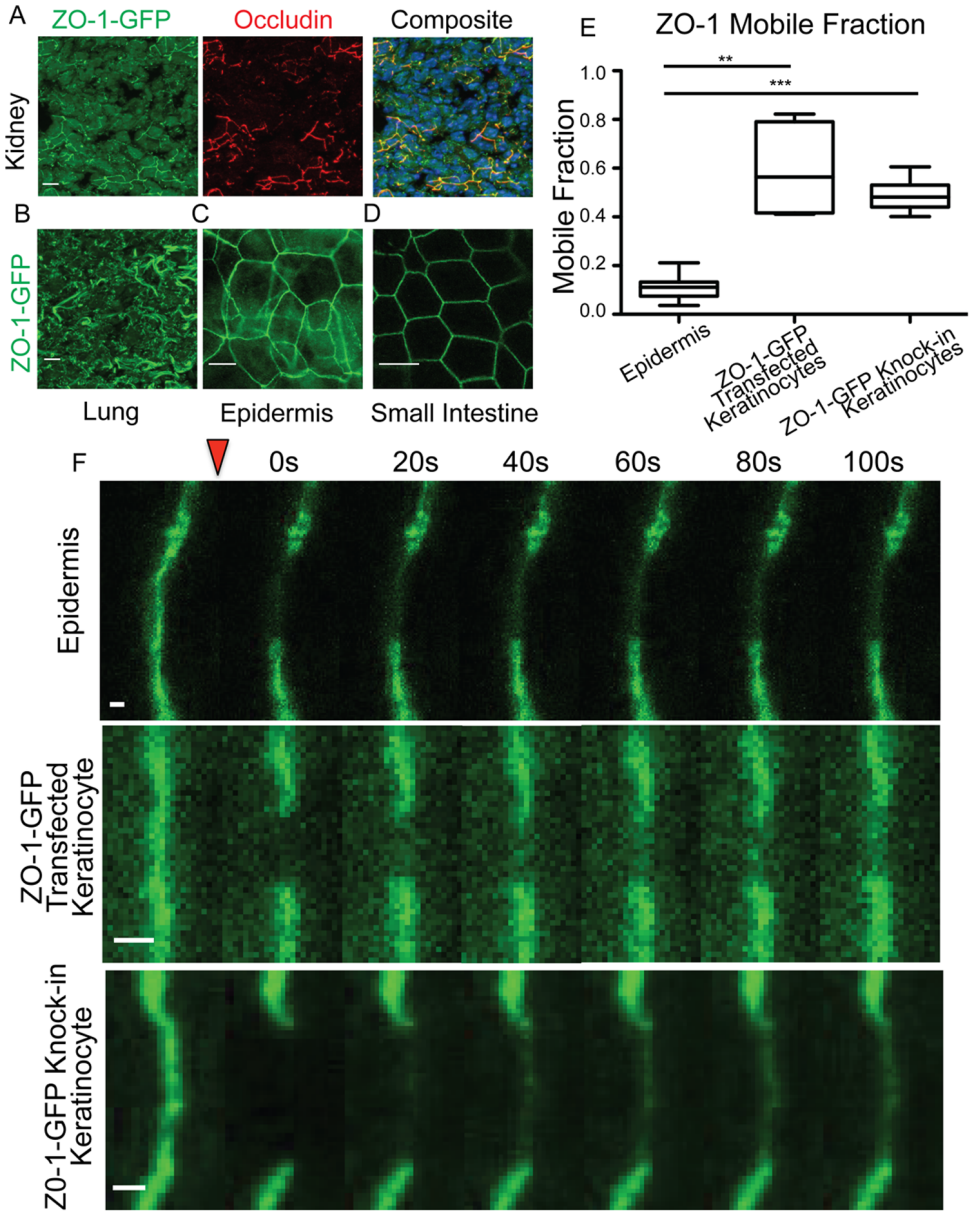
ZO-1 GFP exhibits low mobility in epidermis. A–D) Localization of ZO-1-GFP in skin sections taken from a ZO-1-GFP knock-in mouse. Scale bar 10 µm. A) ZO-1-GFP co-localizes with the tight junction protein occludin in kidney tissue sections of adult mouse. DNA is stained blue. B) Tissue section of lung taken from adult mouse. C) Whole mount epidermis of embryonic day 17.5 mouse. Note the ZO-1 signal in distinctive cobblestone pattern at cell-cell junctions in the granular layer. D) Whole mount small intestine taken from adult mouse. E) Mobile fractions from FRAP experiments are plotted. The box represents the 25^th^ to 75^th^ percentile and the whiskers represent the 10^th^ and 90^th^ percentiles. ** p<.005. *** p<.0001. F) Representative kymographs are shown of individual FRAP experiments. The bleach point is indicated by the red triangle. Scale bar 1 µm.

Consistent with this, we found that ZO-1-GFP was expressed and localized to cell-cell junctions in all epithelial tissues examined as well as in the heart and other cell types that express ZO-1. Appropriate co-localization with the transmembrane tight junction protein occludin can be seen in the kidney (Figure 1A) and tight junction localization can be seen in lung tissue (Figure 1B). Whole mount imaging of the epidermis and the small intestine revealed the organized polygon structure of ZO-1 at cell-cell boarders (Figure 1C,D). ZO-1 can be seen in a few overlying cell layers in the upper granular layers of the epidermis, while the small intestine, as a simple epithelium, has only a single layer of tight junctions.

### Slow turnover of ZO-1-GFP in epidermis as compared to cultured keratinocytes

Having established this mouse strain, we began to examine the dynamics and turnover of ZO-1 at tight junctions in the epidermis using Fluorescence Recovery After Photobleaching (FRAP) experiments. We placed embryonic day 17.5 (e17.5) embryos in a glass-bottomed dish with media, and imaged on a confocal microscope with an environment control chamber, allowing for maintenance of temperature at 37°C and CO_2_ at 5% to maintain embryo viability. A custom built trapping device ensured the epidermis made contact with the glass, allowing for effective imaging. Surprisingly, ZO-1-GFP did not show a robust recovery after photobleaching, with a mean mobile fraction of 11% five minutes after bleaching, indicating that almost 90% of ZO-1 remains immobile at the cortex (Figure 1E). This result was unexpected as ZO-1 turnover in cultured MDCK cells is both more rapid and more complete [20]. A representative kymograph demonstrates the minimal recovery at epidermal tight junctions (Figure 1F). The difference between the epidermis and MDCK cells could be explained by cell type differences or the tissue context. We therefore assayed the turnover of ZO-1-GFP when transfected into cultured keratinocytes. These cells showed a rapid recovery with a large observed mobile fraction (Figure 1E,F). Therefore, ZO-1 shows a substantial decrease in mobility in the epidermis compared to cultured cells.

The observed difference in mobile fractions between intact epidermis and cultured keratinocytes could be due to overexpression artifacts upon transfection or could reflect differences in turnover between cultured cells and tissue. To distinguish between these, we generated cultured keratinocytes from the ZO-1-GFP knock-in mouse to avoid the possible effects of overexpression and transfection. In these cells, ZO-1 was highly mobile, with a mobile fraction comparable to transfected keratinocytes (Figure 1E). We conclude from these studies that the levels of ZO-1 do not dramatically affect the turnover rate at the cell cortex in cultured keratinocytes, and, that ZO-1 is much more stable in intact epidermis than in cultured keratinocytes.

A simple explanation for the limited observed recovery of ZO-1 in the epidermis could be that, under the conditions we used for imaging tissues, the cells were inviable. This is unlikely to be the case as under similar conditions we have observed the microtubule dynamics to be comparable to those in cultured cells (data not shown).

### Slow turnover of E-cadherin in epidermis as compared to cultured keratinocytes

**Figure 2:**
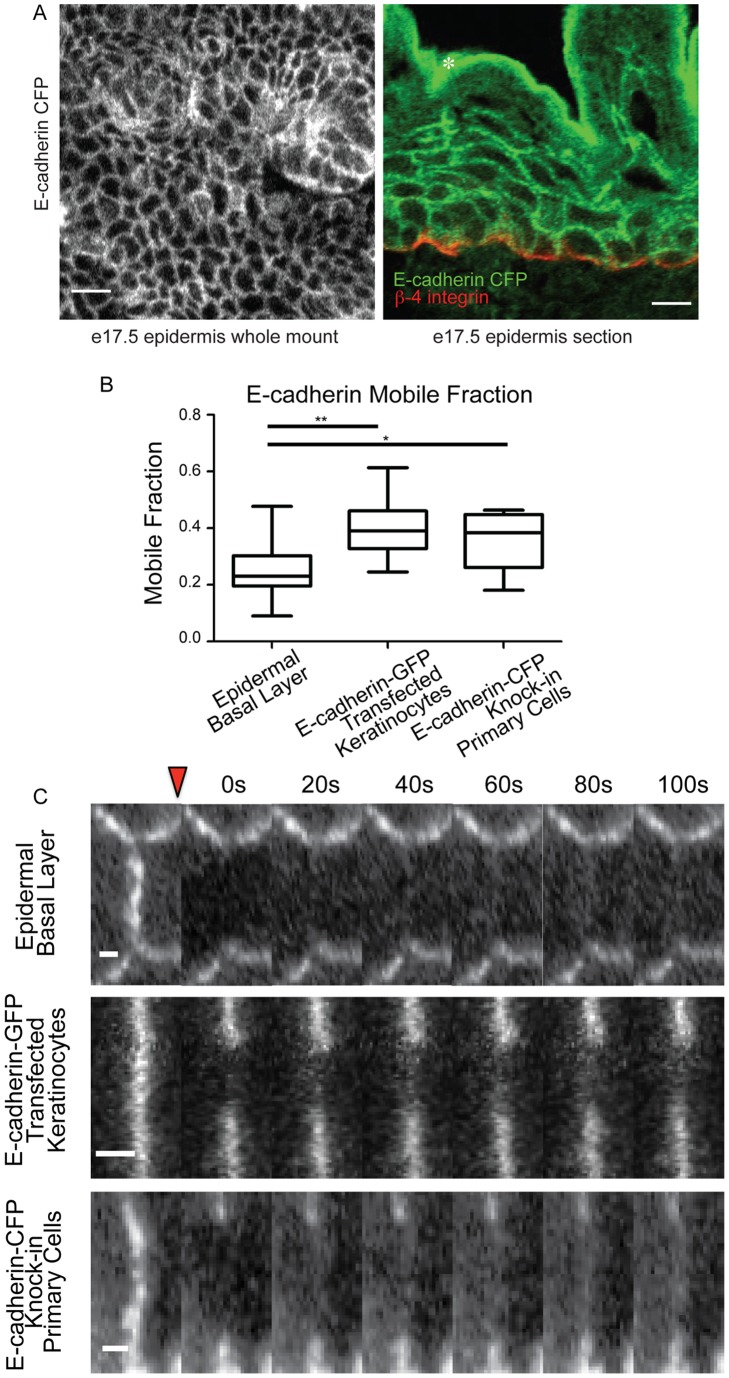
E-cadherin shows limited recovery at adherens junctions in tissue. A) Wholemount (left) and cross section view (right) of E-cadherin-CFP epidermis. In the cross section, β4-integrin staining was used to define the basement membrane (red). Scale bars are 10 µm. B) Mobile fractions from FRAP experiments are plotted. The box represents the 25^th^ to 75^th^ percentile and the whiskers represent the 10^th^ and 90^th^ percentiles. * p<.02. ** p<.001. C) Representative kymographs are shown of individual FRAP experiments. The bleach point is indicated by the red triangle. Scale bar 1 µm.

The dramatic difference in ZO-1 turnover in tissue prompted us to examine the turnover of adherens junction proteins. We have previously measured the mobile fraction of the homophilic transmembrane protein E-cadherin in cultured keratinocytes to be around 40% [4]. To observe E-cadherin dynamics in intact tissue, we took advantage of the previously generated E-cadherin-CFP knock-in mouse [18]. These mice allowed visualization of E-cadherin in the epidermis by both wholemount analysis and in tissue sections (Figure 2A). With E-cadherin, we were interested in the dynamics in the basal progenitor cells of the epidermis, rather than in the upper granular layers where tight junctions and ZO-1 are found. As the basal cells are proliferative, they are more likely to have dynamic junctions. We therefore collected backskin samples from e17.5 embryos, separated the epidermis from the dermis, and plated the isolated epidermis on glass-bottom dishes to allow for the direct imaging of E-cadherin dynamics. Cells remain viable under these *ex vivo* conditions, as we have imaged dynamic microtubules under these conditions using a different transgenic line (data not shown).

Similarly to ZO-1 in the suprabasal epidermis, E-cadherin in the basal layer showed reduced mobility compared to E-cadherin in cultured keratinoctyes (Figure 2B). Representative kymographs demonstrate the difference in recovery dynamics (Figure 2C).

In order to determine if the observed stabilization of E-cadherin was dependent on the tissue architecture of the epidermis, we isolated primary cells from the epidermal basal layer of E-cadherin-CFP mice. After two days in culture to allow the cells to grow confluent and form adhesions, we analyzed the recovery at adherens junctions in these primary cells. They exhibited a recovery similar to that of the transfected keratinocytes, with a significantly higher mobile fraction than that observed in the epidermis (Figure 2B). A representative kymograph demonstrates this recovery (Figure 2C).

**Figure 3:**
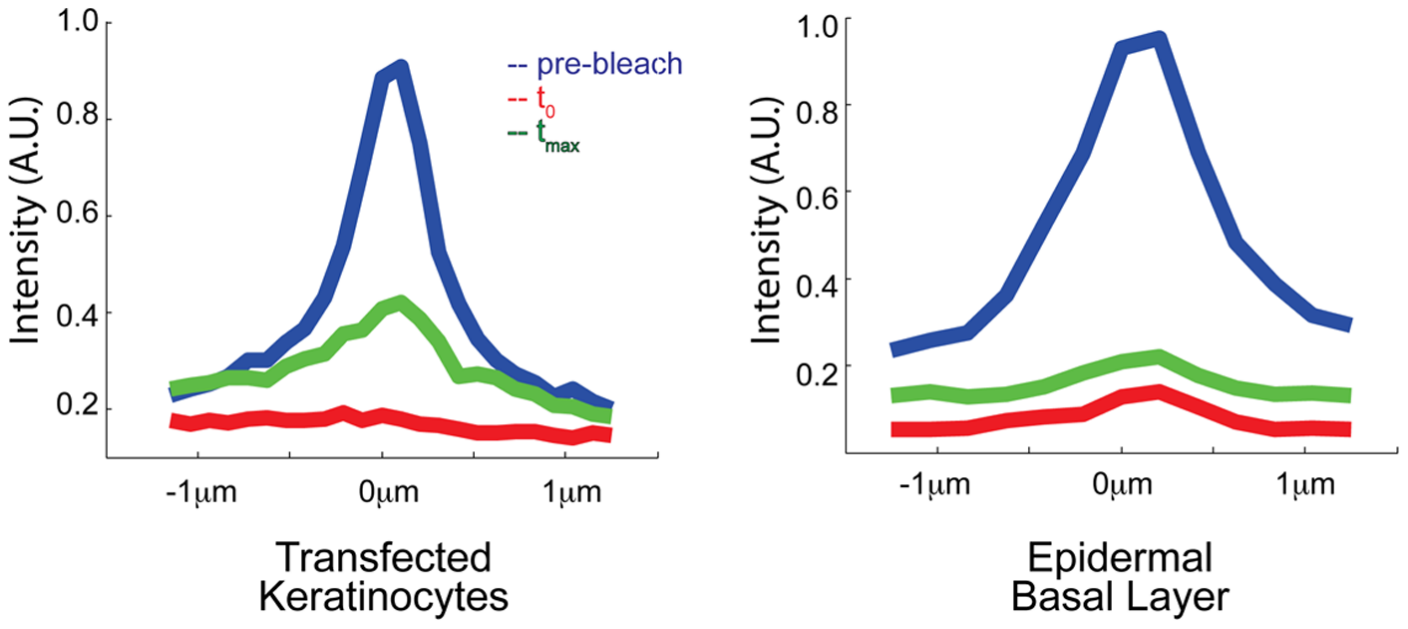
Intensity profiles of fluorescence recovery. Graphs show the average intensity profile across bleached junctions in E-cadherin-GFP transfected keratinocytes and in the basal layer of the epidermis from E-cadherin-CFP e17.5 embryos. Time points plotted are directly before bleaching (pre-bleach), directly after bleaching (t_0_), and at maximum intensity recovery within five minutes (t_max_). Each graph shows the average data from n>8 junctions for each condition. Intensity is displayed in arbitrary units for each condition.

While E-cadherin in the basal layer of the epidermis demonstrated a mobile fraction of approximately half of that observed in transfected keratinocytes, this mobile fraction may be an overestimate of the recovery at the junction. To quantify the junction-specific fluorescence recovery, we calculated the combined average intensity profile of all junctions in each condition at time points before bleaching, immediately after bleaching, and at maximal recovery within five minutes (Figure 3). E-cadherin in transfected keratinoctyes demonstrates a strong junction-specific turnover, seen by the clear peak in the maximal recovery curve (green colored curve in Figure 3). In basal cells, however, the fluorescence recovery over the junction is fairly uniform and can largely be attributed to simple diffusion of the cytoplasmic pool, rather than turnover at adherens junctions.

### Desmoplakin I-GFP shows low turnover both in epidermis and cultured keratinocytes

**Figure 4:**
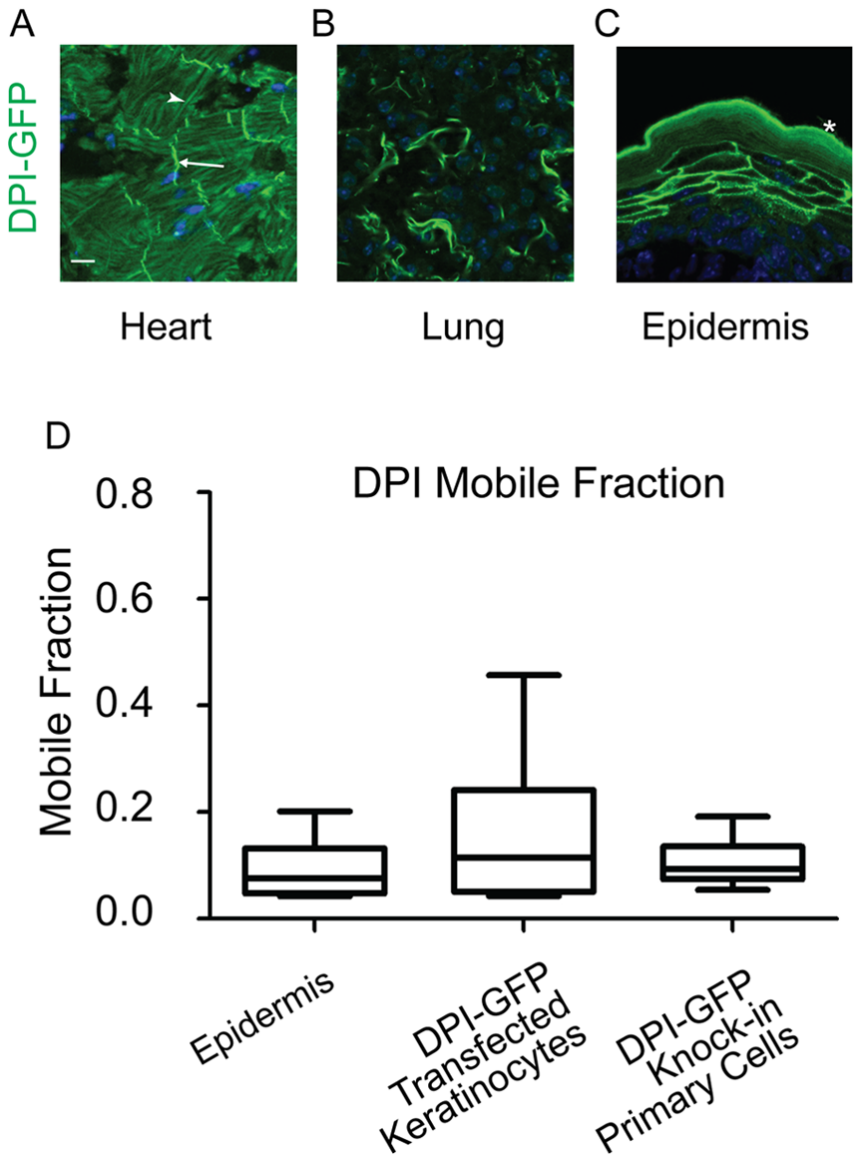
DPI-GFP is stable at desmosomes in both epidermis and in cultured keratinocytes. A–C) Tissue sections from the adult BAC transgenic DPI-GFP mouse. DNA is stained in blue. Scale bar 10 µm. A) DPI-GFP localization in heart tissue section. Note the strong bands of signal at intercalated discs (arrow). There is strong autofluorescence from thick actin bundles in cardiac muscle fibers (arrow head). B) DPI-GFP localization in lung tissue section. C) DPI-GFP localization in epidermal tissue section. Asterisk labels autofluorescence of cornified layer. D) Mobile fractions from FRAP experiments are plotted. The box represents the 25^th^ to 75^th^ percentile and the whiskers represent the 10^th^ and 90^th^ percentiles.

The third type of epithelial adhesion structure is the desmosome. To date, no tools have been generated to study desmosome dynamics in mammalian tissues. Additionally, as invertebrates do not have desmosomes, they have yet to be observed in any live organism. To observe desmosome dynamics in tissue, we generated BAC-transgenic mice that expressed GFP tagged desmoplakin isoform I (DPI-GFP) with about 100 kB of control elements from the DP promoter. This Bac transgenic DPI-GFP mouse showed proper DPI-GFP localization in the heart, lung, skin and endothelia (Figure 4A–C, and data not shown). Sections of the heart showed strong localization at intercalated discs, distinctive junctions found in cardiac muscle tissue (Figure 4A). There was also clear localization to epidermal cell-cell contacts (Figure 4C). This mouse should prove a useful tool for future studies of desmosome dynamics, especially in mutant and pathological settings.

In cultured keratinocytes, DPI-GFP is highly stable, with little protein turnover [21]. Using embryos from these mice, we determined DP to have a very low mobility in the epidermis as well (Figure 4D). DPI in isolated primary cells was also quite stable (Figure 4D). As DP displays a low mobility in cultured keratinocytes, there was no conspicuous difference between turnover at desmosomes in the epidermis and in keratinocytes. These data suggest that using cultured keratinocytes as a model for desmosome dynamics and turnover is appropriate.

## Discussion

We have used fluorescently labeled proteins in mouse epidermis to investigate the dynamics of proteins from three different junctions. The tight junction protein ZO-1, the adherens junction protein E-cadherin, and the desmosomal protein desmoplakin were all observed to be remarkably stable within the epidermis. The limited and slow recovery observed in tissue contrasts with the observed dynamics of ZO-1 and E-cadherin in both keratinocytes and primary cells, which both demonstrate significantly higher mobility.

Recent studies in *Drosophila* have begun to explore *in vivo* dynamics of junctional proteins. *Drosophila* DE-cadherin and the beta-catenin homolog armadillo exhibit significantly lower mobile fractions in late-stage embryos compared to early embryos [22]. Additionally, increasing tensile forces can result in a rapid increase in integrin stability at *Drosophila* myotendious junctions [23]. These observations demonstrate that the mobility of proteins within junctions are not constant but rather vary as tissues develop and adapt to external and internal changes. In a previous study, ZO-1 turnover was measured in small intestine of a mouse and determined to recover at levels similar to those seen in cell culture [24]. It is likely that the lack of recovery that we observed reflects a tissue specific stabilization of junctions in the epidermis.

The increased turnover of adherens and tight junction proteins in cultured keratinocytes as compared to intact epidermis suggests that these adhesions are in different states in these different environments. Junctions in cultured cells may resemble those in more dynamic tissues, such as during migration or during wound healing events, in which junctions are being remodeled. This is consistent with previous reports that demonstrate upregulation of wound healing genes in cultured keratinocytes [25]. Our data indicate that junctions within tissues may have additional mechanisms of stabilization that are not modeled by cultured cells. It will be important to fully understand how these protein dynamics are regulated and how the tissue architecture contributes to junction stability and function.

Turnover at the junction can be accomplished through two main mechanisms, either through lateral diffusion of junctional proteins, or through exchange with a cytoplasmic pool. Previous studies of both ZO-1 [20] and DE-cadherin [22] have concluded that turnover of these proteins at the junction is predominantly through exo- and endocytotic exchange with a cytoplasmic pool. Stabilizing these proteins would require a decrease in the rate of removal at the membrane or a decrease in the rate of delivery to the membrane. These changes could be realized through biochemical differences such as posttranslational modifications to junctional proteins that decrease their internalization [10], [11]. Further work must address the exact mechanisms of this stabilization.

By creating a knock-in ZO-1 GFP mouse and Bac transgenic DPI-GFP mouse as well as a method for imaging the epidermis of live embryos, we have developed new ways to probe epidermal junction dynamics and function. These tools will allow us to better investigate cell-cell junctions and study their formation, regulation, and stabilization.

## Materials and Methods

### Mouse Studies

All mouse studies were performed with approval from Duke Institutional Animal Care and Use Committee (IACUC).

### Tissue sections

10 µm thick sections were fixed in 4% paraformaldehyde. For staining with occludin, a rabbit anti-occludin (Abcam) antibody was used. Sections were imaged with a microscope (AxioImager Z1; Carl Zeiss) with Apotome attachment, 40×1.3 NA EC Plan NEOFLUAR objective, AxioCam MRm camera, and Axiovision software (Carl Zeiss).

### Cell Culture FRAP experiments

Wildtype keratinocytes were grown at 37°C and 7.5% CO_2_ in E low Ca^2+^ media. Cells were plated onto 35 mm glass-bottomed dishes (MatTek Corporation) and transfected using TransIT-LT1 transfection reagent (Mirus) with ZO-1-GFP, E-cadhein-GFP, or DPI-GFP plasmids. When confluent, cells were switched to high calcium (1.2 mM) E media to promote adhesion formation for 16–20 hours. Cells were imaged using an LSM 710 confocal microscope (Carl Zeiss) with ZEN imaging software and a temperature control chamber set to 37°C and 5% CO_2_. A 63×1.4 NA oil immersion objective was used. Percent recovery was calculated as previously described [21]. The fluorescence intensity of each region was first adjusted by subtracting the background intensity and then normalized to the intensity at the initial time point. The mobile fraction was then calculated as (I_max_ –I_0_/(1 – I_0_) where I_max_ is the maximal fluorescence recovery and I_0_ is the initial fluorescence intensity immediately after photobleaching [20]. All FRAP experiments were conducted on at least two separate days and at least thirteen individual junctions were analyzed under each condition.

### Epidermis FRAP experiments

Embryos were taken at embryonic day e17.5, placed in 35 mm glass-bottomed dishes, and immersed in warmed high calcium E media. A trapping device consisting of a flexible membrane sandwiched between two brass rings was used to ensure the embryo was pressed against the glass. The junctions were then imaged and analyzed with the same process as the cultured keratinocytes.

### Basal layer Isolation

Backskins were removed from e17.5 embryos and placed in a 1∶1 dispase:PBS solution at 37°C for 1 hour to separate the epidermis from the dermis. The epidermis was then placed basal side down into a 35 mm glass-bottomed dish. A small weight was placed on top of the section to ensure the cells made contact with the glass. The epidermis was then immersed in warmed high calcium E media and imaged and analyzed with the same process as the cultured keratinocytes.

### Primary cell FRAP experiments

Backskins were removed from e17.5 embryos and placed in a 1∶1 displase:PBS solution at 37°C for 1 hour. The epidermis was removed and then placed in a 1∶1 trypsin:versene solution at 37°C to separate off individual cells. Cells were then plated onto a fibronectin (10 µg/mL) coated 35 mm glass-bottomed dish and grown in E media at 0.3 mM Ca^2+^. When confluent the cells were switched to high calcium (1.2 mM) E media for 16–20 hours. The junctions were then imaged and analyzed with the same process as the cultured keratinocytes.
